# Sensory profiles in older adults with orthopedic conditions during quiet stance: a cross-sectional study

**DOI:** 10.1186/s11556-025-00368-9

**Published:** 2025-02-26

**Authors:** Marine Brika, France Mourey, Alexandre Kubicki

**Affiliations:** 1 https://ror.org/03pcc9z86 grid.7459.f 0000 0001 2188 3779 grid.7459.f, Université Marie et Louis Pasteur, Besançon, France; 2 https://ror.org/02vjkv261 grid.7429.8 0000 0001 2186 6389 Université Marie et Louis Pasteur, INSERM, UMR 1322 LINC, F-25000 Besançon, France; 3 https://ror.org/03k1bsr36 grid.5613.1 0000 0001 2298 9313 grid.5613.1, University of Burgundy, Dijon, France; 4 https://ror.org/02dn7x778 grid.493090.7 0000 0004 4910 6615 INSERM UMR1093-CAPS, Université Bourgogne Franche-Comté, UFR des Sciences du Sport, F-21000, Dijon, France

**Keywords:** Sensory reweighting, Older adults, Podal dependance, Cervical proprioception

## Abstract

**Background:**

Pathological aging can impair sensory information, leading to postural control disorders in older adults. Compensatory sensorial mechanisms are emerging to preserve balance function. The objective of the study was to identify sensory profiles in functionally impaired older adults, and determine if they are linked to the frequently observed cervical proprioceptive disorders in this population.

**Methods:**

Fifty-one older adults (76.9 ± 7.6 years) were divided into 2 Functional Groups (FG-/FG+) according to a composite score that included 3 variables (gait speed, grip strength and fear of falling). All the participants completed the modified clinical test of sensory interaction on balance (m-CTSIB) and the cervical joint sense position error (CJPSE) test. Exploratory factor analysis was used to identify common factors among the variables. Pearson correlation was used to examine relationships between variables.

**Results:**

As expected, conditions 2 and 3 of the m-CTSIB were both challenging to balance, whereas condition 4 was too difficult for several patients. Factor analysis revealed that the stabilometric variables were grouped together in factor one, and proprioceptive performance (CJPSE) and the mean CoP velocity in m-CTSIB condition 3 formed another second factor. Moreover, a significant correlation was highlighted between stability in Condition 3 and CJPSE in the FG-.

**Conclusion:**

Our results revealed the predominance of both visual and podal information in functionally impaired adults to control their posture. We speculate that the observed podal preference could be consecutive to a less efficient cervical proprioceptive system.

## Introduction

Postural control is influenced by the availability of sensory inputs, including visual, proprioceptive, and vestibular information [1]. The dynamic reweighting of these sensory inputs ensures the optimization of motor and postural performance [1, 2]. This sensory integration becomes particularly relevant during aging, as it is often disrupted by impairments in the musculoskeletal system [3, 4]. Aging is a complex process that leads to declines in visual acuity, proprioception and vestibular reflexes compounded by degenerative changes in the neuromuscular system [5–7]. To counteract these age-related effects, older adults often develop compensatory mechanisms to preserve balance [8]. For instance, increased reliance on visual information is a well-documented adaptation in both normal and pathological aging [9, 10]. Other authors reported that the impact of proprioception information increases with age due to the degradation of other sensory inputs [3]. During quiet stance, the feet act as the sole interface with the ground and play a crucial role in sustaining balance [11, 12]. However, similar to other proprioceptive receptors, the efficiency of podal receptors may decline with age.

This alteration in sensory information can compromise postural control, ultimately impacting the functional capabilities of older adults. In this population, such declines often contribute to a state of frailty, defined as a reduction in the intrinsic individual’s capacities across physiologic systems and heightened vulnerability to environmental stressors [13]. The relationship between sensory impairments and frailty is well established in the literature [14, 15]. For example, chronic pain in older adults is correlated with frailty [14] while de Mettelinge et al. have suggested a causal link between reduced cervical proprioception and an increased risk of falls [15].

Given the diversity of age-related impairments, compensation is dependent on individual history and preference [16]. Therefore, the primary objective of this study was to analyze the sensory profiles of older adults with orthopedic conditions focusing on the three principal sensory inputs (plantar proprioception, visual, and vestibular). Our recent findings demonstrated that frail patients are similarly perturbed by the closed eyes and foam surface conditions [12]. We hypothesized that these results would be replicated in this study population. Additionally, we aimed to explore potential associations between sensory preferences and cervical proprioceptive deficits which are prevalent in this population. This potential correlation could explain an internal compensation substituting a less efficient proprioceptive system.

## Methods

### Participants

This study is an observational cross-sectional study. Ethical approval was obtained from the University Ethics Committee in September 2021 (CERUBFC-2021-11-09-036). The study was performed in accordance with the ethical practices outlined in the Declaration of Helsinki (1964).

Fifty-one volunteer older adults (76.9 ± 7.6 years, 13 males and 37 females) were recruited by a physiotherapist from a readaptation center located in Montbéliard (France) between February 2022 and June 2023.

All the participants were aged 65 years and over and presented with some orthopedic disabilities (hip or knee arthroplasty). However, all participants presented a corrected vision deficit and an ankle muscle strength > 3/5 on manual muscle strength assessment. None of them had diabetes, vestibular pathology or major neurocognitive disorders. Written informed consent was obtained from each participant.

The participants’ anthropometric data (gender, age, and body mass index) were collected. Fall history from the previous 6 months and the presence (or absence) of fear of falling (assessed using the Short-FES scale) [17] and neck pain (visual analog scale) were also recorded. The range of ankle dorsiflexion was measured for each participant, as were their grip strength and gait speed. Ankle dorsiflexion range of motion was measured bilaterally using a inclinometer [18]. Grip strength was assessed using a Jamar^®^ dynamometer, following established guidelines [19]. Gait speed was assessed over a 10-meter distance, with time measured at self-selected pace [20].

The participants were divided into two functional groups (FG-/FG+) according to a composite score that included 3 variables (gait speed, grip strength and fear of falling).

### Calculation of the composite score for functional ability

The participant’s gait speed was expressed relative to the maximum gait speed of our sample (m.s^− 1^).

GSpeed’ = (Gait speed × 100)/1.2.

The participant’s grip strength was expressed relative to the maximum grip strength of our sample (kg).

GStrength’: (Grip strength × 100)/46.9.

The composite score was subsequently calculated via the following formula:

Composite score = (Gspeed’+Gstrength’) +/- 10%.

+/- 10% was applied depending on the presence (+) or absence (-) of fear of falling.

### Procedure

The protocol included an evaluation session performed by an evaluator unaware of the participants. All the participants completed the modified Clinical Test of Sensory Interaction on Balance (m-CTSIB) and performed one proprioception test. The exclusion criterion encompassed incomplete stabilometric evaluations, defined as participants completing only 2 out of 3 trials per condition or evaluating only 2 out of 4 specified conditions.

#### Modified clinical test of sensory interaction on balance (m-CTSIB)

The test used a stabilometric platform (Techno concept^®^ with Posturewin software, France). The participants were instructed to stand barefoot on the platform and adopt the reference position: feet shoulder width apart, arms at their sides and gazing straight ahead at a visual cue. Throughout the 20-second data acquisition, the participants maintained stillness while their center-of-pressure (CoP) trajectory was captured at a rate of 40 Hz by the platform’s three strain gauges. The plate level thickness was 0.002 m from the ground. A foam surface (Airex^®^, height 50 cm, width 41 cm, thickness 6 cm, density 55 kg.m^− 3^) was used to disrupt the contribution of podal information to postural regulation.

Four conditions, comprising a measurement block, were assessed: standing reference position with eyes open (condition 1), standing with eyes closed (condition 2), standing on foam with eyes open (condition 3), and standing on foam with eyes closed (condition 4). The participants completed three measurement blocks, separated by breaks between each block. If participants required assistance to maintain balance, e.g., with light support on a chair or the physiotherapist to prevent them from falling, the trial was stopped and not retained.

#### Proprioception tests

After executing the m-CTSIB test, the participants were instructed to perform the (neutral) cervical repositioning test (or cervical joint sense position error) [21]. In the sitting position, participants wore a helmet with a laser pointer and were asked to memorize the neutral reference position, which consists of looking straight ahead with the laser pointing at the center of a target 90 cm away. With the eyes closed, the participants performed active rotational movements in the transverse plane through the full range of motion, from left to right. The objective was to return to the reference position with maximum precision consistently with the eyes closed. Each trial involved measuring the distance from the target’s center to the laser’s arrival point, which was determined when the participant believed that he or she had accurately returned to the reference position.

Three trials were performed, and the results were averaged. In accordance with the same principle, three cervical repositioning tests were also performed after active flexion/extension movement (sagittal plane) [22].

### Data analysis

For the cervical repositioning test, the difference between the starting position (zero) and the point of return in the plane of movement was measured in centimeters and then converted to degrees via the following formula: angle = tan^− 1^ [error distance/90 cm] [23]. Only the absolute error was calculated and defined as the mean of the total deviation from the target, ignoring the positive and negative values [24].

Before conducting the statistical analysis, the normality (Shapiro‒Wilk test, all *p* > 0.01) of each variable was checked. The comparison of fundamental clinical data (shoe size, weight, body mass index, dorsiflexion, performance of cervical proprioception tests) and stabilometric data (CoP mean velocity during conditions 1, 2 and 3 of the m-CTSIB) between the two groups utilized the Mann‒Whitney test. Student’s t test was used to compare the following variables: age, height, walking speed and CoP mean velocity during condition 4 of the m-CTSIB. Additionally, a χ² test with Yates correction was performed for the variable “history of falls”.

An exploratory factor analysis based on a matrix of correlated associations was carried out to determine common factors between variables. Considering the nonnormality of several variables, the method used for factor extraction was the “principal axis factor” procedure. Next, Bartlett’s test was used to verify the hypothesis of sphericity, and the Kaiser‒Meyer‒Olkin (KMO) test was used to check the adequacy of the sampling. The number of factors was determined via the parallel analysis method, which considers the number of factors whose eigenvalues are greater than those obtained with random data. The sum of the square loadings and the proportion of variance were subsequently calculated. The applied rotation method was either oblique or orthogonal, depending on the factor loading coefficient.

If stabilometric variables seem to be linked with repositioning variables, and in the case of normality and homogeneity of these variables, a characterization of the relationship was performed via the Pearson coefficient.

For all the statistical analyses, the significance level was set at *p* < 0.05. Statistical analyses were performed via JASP^®^ software (Version 0.16.3, JASP Team, University of Amsterdam, Netherlands).

## Results

Within the sample of 51 participants (total group, TG), 3 participants were excluded because the mean value of one of their clinical or stabilometric variables exceeded 2 times the standard deviation. More specifically, 2 participants were excluded on the basis of the weight variable, and 1 participant was excluded on the basis of the mean velocity of the center of pressure variable in condition 3. Consequently, 48 participants were included in the subsequent statistical analysis.

### Participants’ characteristics

On the basis of the anthropometric data, both groups of participants were similar in all aspects. In terms of the clinical data, there was a significant difference in the walking speed between the two groups. Compared with the FG + group, the FG- group walked significantly more slowly over a distance of 10 m (*p* < 0.001). Similarly, a significant difference was observed in the composite score between the two groups (*p* < 0.001).

### Exploratory factor analysis

**Fig. 1:**
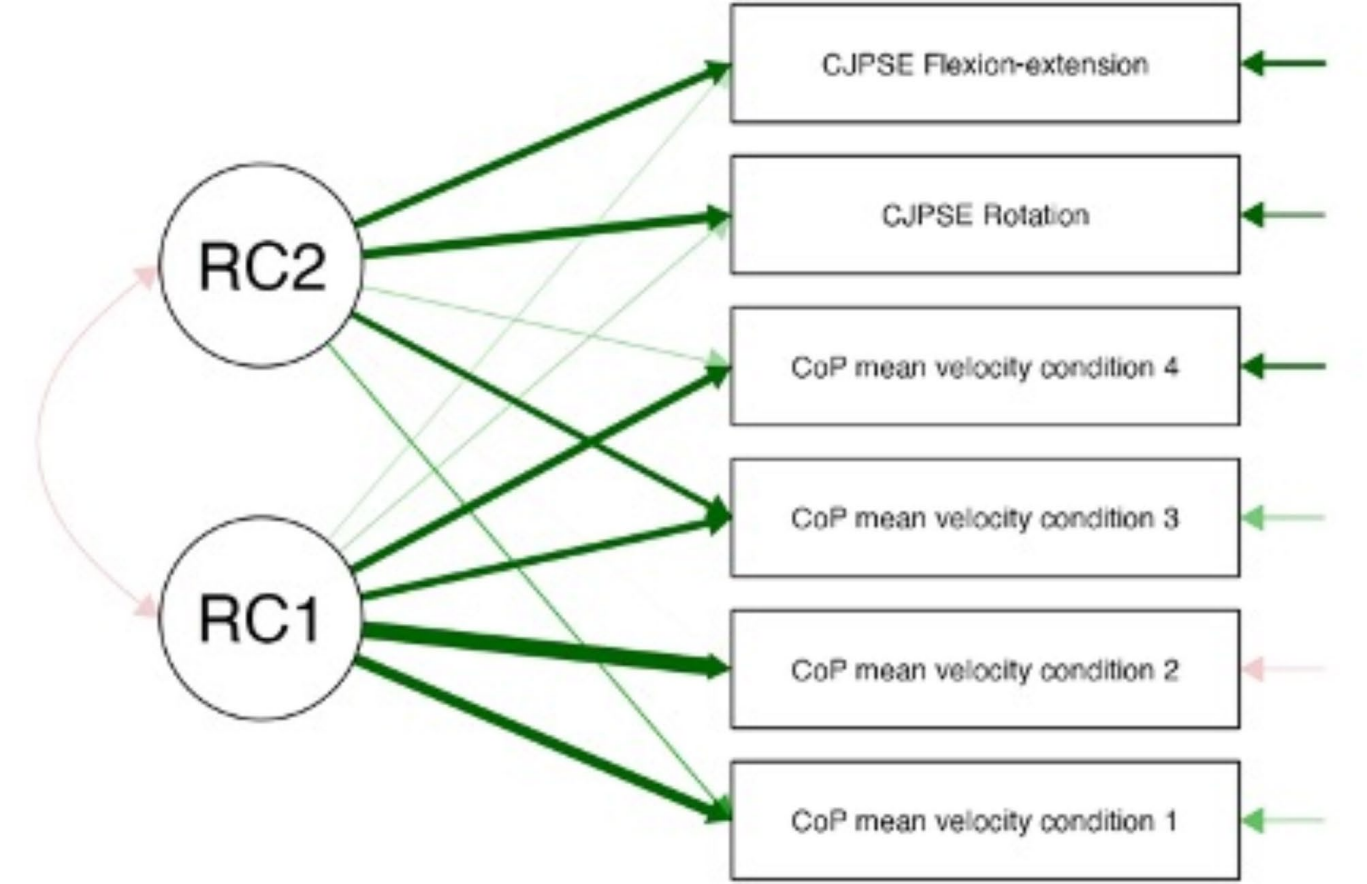
Path diagram following the factorial analysis. Red arrows indicate a negative correlation with the factor. The green arrows indicate a positive correlation with the factor. The thickness of the arrows indicates the degree of importance of the variable in relation to the factor and the degree of error importance

The variables selected for exploratory factor analysis were weight, composite score, proprioception test performance (CJPSE flexion-extension and CJPSE rotation) and stabilometric variables (mean CoP velocity in the 4 conditions of the m-CTSIB) (see Fig. 1).

In the initial phase of assessing the feasibility of hypothesis testing through factorial analysis, Bartlett’s sphericity test was significant (*p* < 0.01), indicating sufficient correlations between variables. All the variables tested with the KMO test (factorial solution adequacy) presented values exceeding 0.6 (mean MSA = 0.68).

In the second phase, a data rotation procedure was performed. Prior to the rotation procedure, exploratory factor analysis revealed 2 factors. The sum of the square loadings was 3.196 and 0.971 for factor 1 and factor 2, respectively. Factor 1 explained 53% of the variance in these variables, whereas factor 2 explained 16% of the variance, resulting in a combined explanation of 69% of the variance explained by the 2 factors.

**Fig. 2:**
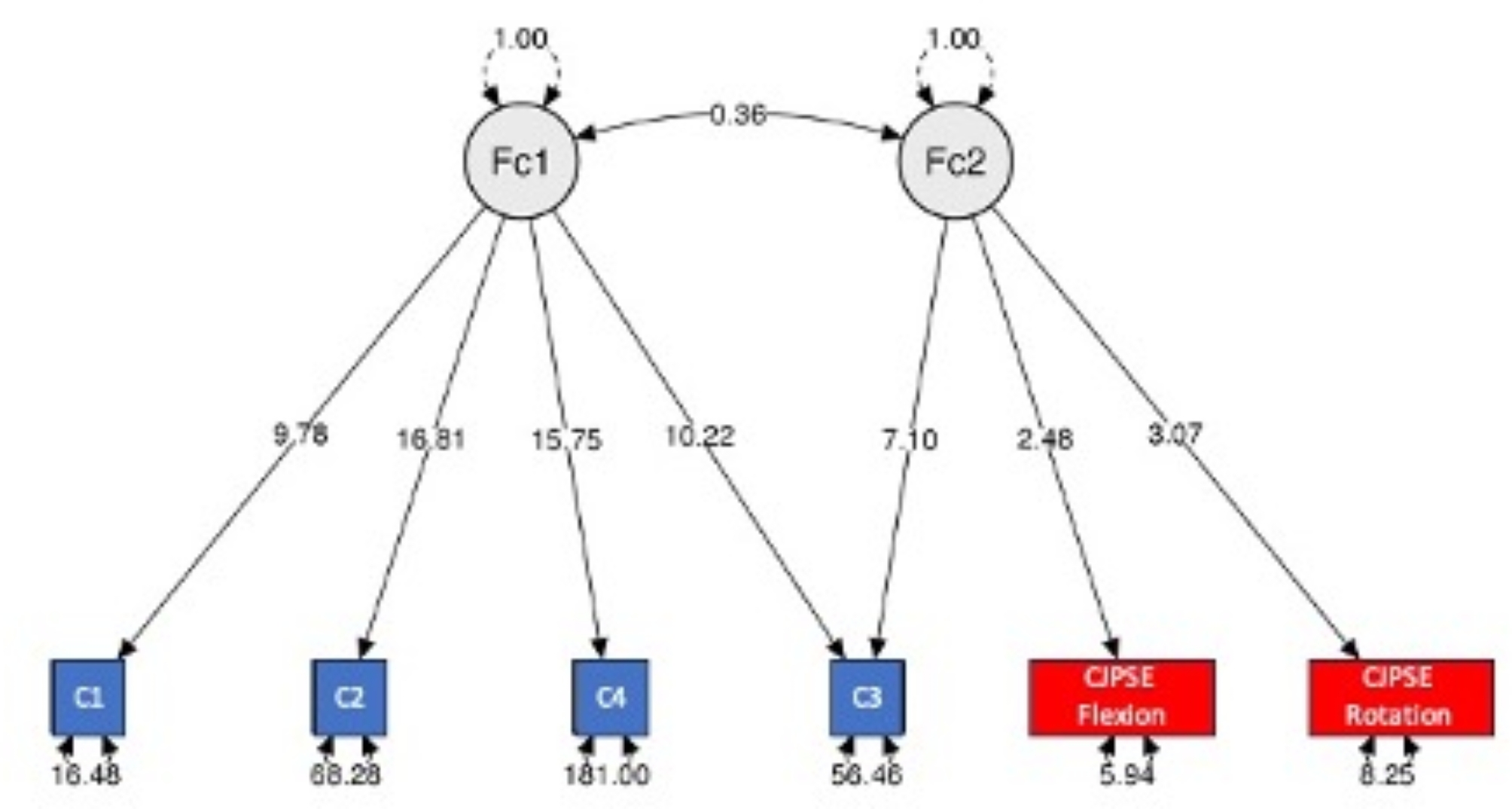
Model plot following confirmatory factor analysis The numbers written on the black lines correspond to the weight of each variable in the associated factor. The numbers below the squares indicate the conditions of the m-CTSIB (C1, C2, C4, C3), and proprioceptive performance (CJPSE flexion, CJPSE rotation) represents the values of residual variances. The notation 0.36 between the two factors corresponds to the covariance weight of the factors

An orthogonal rotation was chosen because the correlation value of the factors in oblique rotation was 0.15. After the rotation procedure, the same 2 factors remained discernible. Factor 1 included all the stabilometric variables, whereas factor 2 included proprioceptive performance (CJPSE flexion-extension and CJPSE rotation) and the mean velocity of CoP on foam with eyes open (Condition 3 of the m-CTSIB) (see Fig. 2).

After the rotation procedure, the sum of the square loadings was 2.650 and 1.489 for factor 1 and factor 2, respectively. Factor 1 explained 44% of the variance in these variables, whereas factor 2 explained 25% of the variance, resulting in a combined explanation of 69% of the variance explained by the 2 factors (Table 1).

**Table 1:** Weights of each variable in their respective factors after rotation (note: the applied rotation method is varimax)

	Factor 1	Factor 2	Uniqueness
**CoP mean velocity condition 2**	1.038		-0.078
**CoP mean velocity condition 1**	0.795		0.244
**CoP mean velocity condition 4**	0.692		0.496
**CoP mean velocity condition 3**	0.659	0.594	0.213
**CJPSE Rotation**		0.736	0.440
**CJPSE Flexion-extension**		0.667	0.546

### Proprioception tests

#### Cervical repositioning rotation test

For the entire sample, the average performance in the rotation cervical proprioception test was 6.1 ± 4.3°. Within this sample, the mean performance for the FG- sample was 7.5 ± 5.0°, and that for the FG + sample was 4.7 ± 2.4° (*p* < 0.05; please refer to Table 2).

**Table 2:** Patient characteristics and clinical outcomes (mean ± standard deviation or number) for the GF- and GF + groups. For condition 4 of the m-CTSIB, fewer participants were able to perform the test. NA = not applicable

Variable	TG (*n* = 48)	FG- (*n* = 24)	FG+ (*n* = 24)	t/W test value	Significance FG+/FG-	Effect size
Composite score (%)	57.4 ± 16.0	44.7 ± 6.2	71.9 ± 13.2	-9.73	*p* < 0.001	-2.84
Age (years)	77.5 ± 7.3	78.6 ± 7.8	75.2 ± 7.1	1.55	*p* = 0.10	0.45
Height (cm)	162.4 ± 7.5	162 ± 6.1	163.7 ± 9.0	-0.40	*p* = 0.44	-0.12
Weight (kg)	69.0 ± 12.2	67 ± 11.6	74.6 ± 18.0	228.00	*p* = 0.10	-0.17
Body Mass Index	26.1 ± 4.4	25.5 ± 4.5	27.7 ± 5.9	0.54	*p* = 0.20	-0.09
Feet size	39.5 ± 2.0	39.6 ± 1.7	39.7 ± 2.4	277.50	*p* = 0.87	0.01
Ankle dorsiflexion (°)	12.6 ± 5.5	11.9 ± 5.8	13.5 ± 5.1	0.58	*p* = 0.34	-0.09
Neck pain (number participants)	3	3	0	NA	NA	NA
Fall history (last 6 months)	23	16	7	NA	*p* = 0.01	NA
Fear of falling	17	10	7	NA	*p* = 0.49	NA
Gait speed (m.s^− 1^)	0.67 ± 0.17	0.54 ± 0.1	0.80 ± 0.1	-7.58	*p* < 0.001	-2.21
Cervical rotation repositioning initial test (°)	6.1 ± 4.3	7.49 ± 5.1	4.49 ± 2.41	366.50	*p* = 0.03	0.33
Cervical flexion repositioning initial test (°)	5.6 ± 3.5	6.78 ± 4.1	4.74 ± 2.75	352.00	*p* = 0.06	0.33
CoP mean velocity standard position (mm.s^− 1^) *(condition 1 m-CTSIB)*	19.5 ± 10.7	20.6 ± 18.3	17.0 ± 7.7	285.00	*p* = 0.88	0.03
CoP mean velocity eyes closed firm surface (mm.s^− 1^) *(condition 2 m-CTSIB)*	30.3 ± 18.9	30.0 ± 24.1	30.5 ± 12.0	191.00	*p* = 0.07	-0.31
CoP mean velocity eyes open foam surface (mm.s^− 1^) *(condition 3 m-CTSIB)*	31.3 ± 16.4	35.9 ± 20.2	26.6 ± 9.5	336.500	*p* = 0.20	0.219
CoP mean velocity eyes closed foam surface (mm.s^− 1^) *(condition 4 m-CTSIB)*	56.1 ± 19 0.2 (*n* = 31)	51.9 ± 19.8 (*n* = 15)	60.2 ± 18.4 (*n* = 16)	-1.200	*p* = 0.24	-0.431

A significant correlation was observed for the entire sample between proprioceptive performance in rotation and stability on foam with eyes open (*r* = 0.51, *p* < 0.01).

**Fig. 3:**
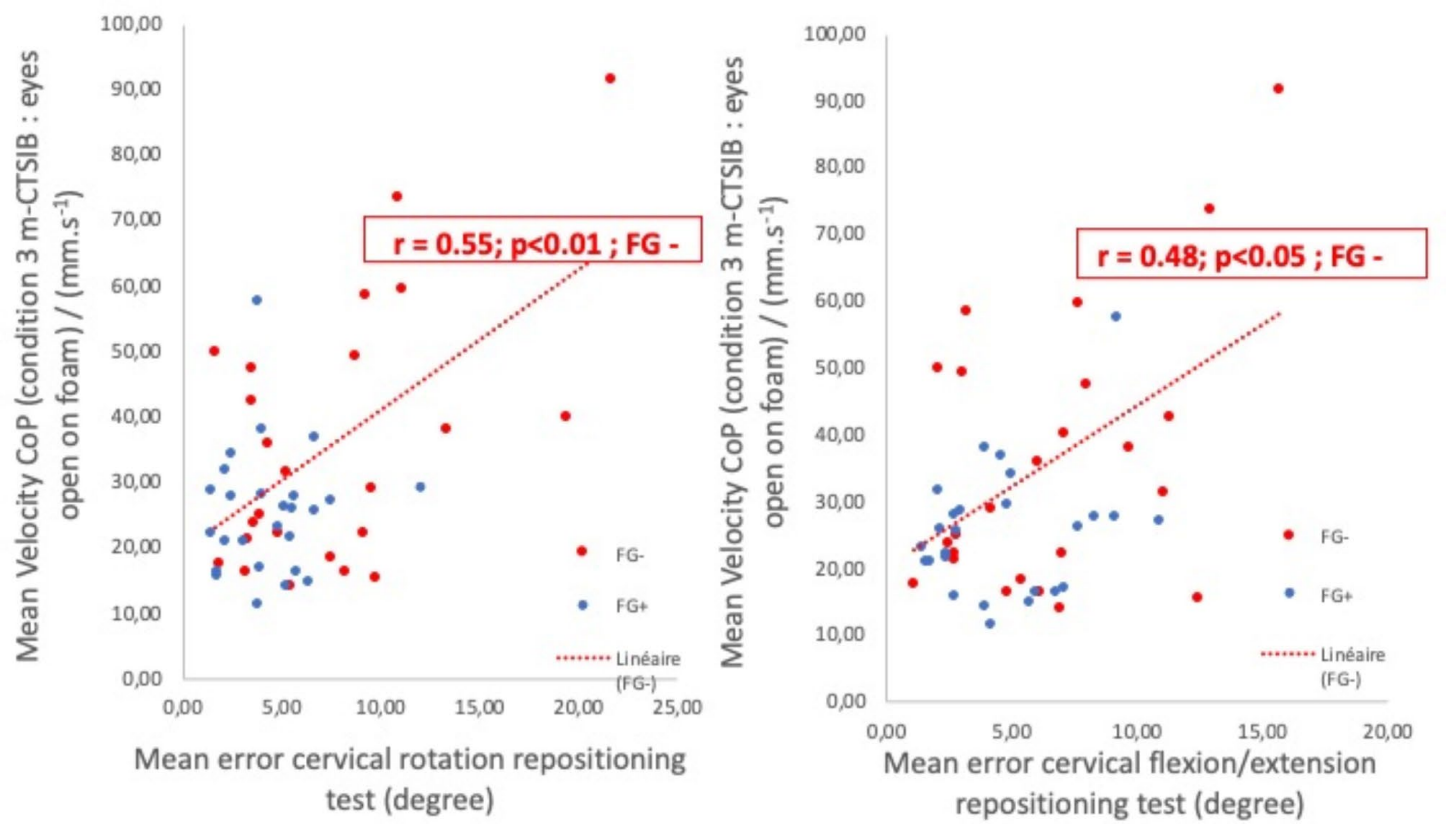
Relationship between the cervical positional error data (x-axis) and the mean CoP velocity displacement speed under Condition 3 (y-axis). The Pearson and associated p values are displayed at the top of the graph. The dotted line represents the regression line in the complete sample

Subsequent correlations were analyzed within each group (FG + and FG-). While no correlation was demonstrated between proprioception test performance and stabilometric evaluation in FG + individuals, a significant correlation was highlighted between stability on foam with eyes open and the precision of rotation repositioning (*r* = 0.55; *p* < 0.01) in FG- individuals (see Fig. 3).

#### Cervical repositioning flexion/extension test

For the entire sample, the average performance in the rotation cervical proprioception test was 6.8 ± 4.1°.

Within this sample, the mean performance for the FG- sample was 6.8 ± 4.1°, and that for the FG + sample was 4.7 ± 2.7° (*p* = 0.06; please refer to Table 1).

A significant correlation was observed in the entire sample between proprioceptive performance in flexion/extension and stability on foam with eyes open (*r* = 0.47; *p* < 0.05).

Subsequent correlations were then analyzed within each group (FG + and FG-). While no correlation was demonstrated between proprioception test performance and stabilometric evaluation in FGs+, a significant correlation was highlighted between stability on foam with eyes open and the precision of cervical flexion/extension repositioning (*r* = 0.48; *p* < 0.05) in FGs (see Fig. 3).

## Discussion

This study aimed to understand the sensory profile of older adults at two different functional levels and examine a potential link between preferential sensory profiles and cervical proprioception. Maintaining a quiet stance without human or technical aid in Condition 4 of m-CSTIB was too challenging for 17 participants. In healthy subjects, this condition seems to be linked to the contribution of the semicircular canals to maintaining disturbed postural balance [25].

Older adults, distinguished by their functional level into two groups (FG + and FG-), exhibited a comparable degree of destabilization under conditions 2 (eyes closed) and 3 (foam) of the m-CTSIB. Our results align with our previous study showing the predominance of both visual and podal information in frail aged adults to control their posture [12]. Notably, the population in our previous study tended to be similar to that in the FG- group in the present study.

The FG + group in this study included relatively robust patients (i.e., nonfrail patients). In this population, the literature suggests the existence of visual reliance [9, 10]. However, Hupfeld’s recent study is in line with our results: their results revealed no age group differences in visual reliance scores between young and older adults. In contrast, they reported a significant difference in condition 3 and concluded that older adults rely more on proprioceptive inputs than young adults do in maintaining balance [26].

Regarding proprioceptive abilities at the cervical level, our study revealed that cervical repositioning tests perform less well in FG- patients than in FG + patients. However, only 3 participants reported neck pain in FG- group. Existing studies have focused on comparing cervical proprioceptive performance between young and healthy older adult. These investigations consistently reveal a decrease in accuracy among older adults when performing this specific task [27, 28].

Our results revealed a difference in the rotation direction of the joint position error test at the functional level (FG- subjects were significantly impaired compared with FG + subjects). With respect to the extension-flexion direction, the between-group analysis revealed a nonsignificant p-shaped trend (0.06). From a biomechanical perspective, rotation is primarily generated by mobility at the C1 and C2 levels, whereas extension/flexion is more related to the lower cervical spine and head-cervical joint at the C0–C1 level. Additionally, the density of joint/muscle receptors appears to be greater in the upper cervical spine [29]. The imprecision of joint and muscle receptors in pathological aging could therefore explain the more pronounced difference in performance in this component of the test.

Interestingly, the factorial analysis revealed 2 factors of 4 variables (factor 1) and 3 variables (factor 2). Factor 2 included proprioceptive variables (CJPSE flexion-extension and CJPSE rotation), and condition 3 included m-CTSIB. This main finding aligns with the results observed in the study conducted by Reddy et al., where a significant correlation (mean Pearson correlation coefficient *r* = 0.7) was identified [30]. This correlation was noted between the functional scores of the Berg and Timed Up and Go (TUG) tests and the error in the cervical repositioning test across both directions [30]. A recent study by Raizah et al. revealed that cervical repositioning test performance was negatively correlated with the Berg score (*r* = -0.7; *p* < 0.001) in older adults with chronic neck pain [31].

Our study revealed a significant correlation within the FG- group, whereas such a correlation was not observed in the FG + group. To explain this result, we hypothesize the existence of “internal” compensation within the somatosensory system. Poor cervical proprioceptive ability might have prompted reweighting in the integration of sensory inputs, resulting in increased reliance on ankle/foot proprioception. Our results are in line with those of the exploratory study of Quek et al., which compared older adults with and without neck pain [32]. These findings indicate that, compared with those without neck pain, older adults with neck pain demonstrated reduced postural stability. These findings suggest that sensory reweighting occurs to engage lower limb proprioception to compensate for deficits in neck proprioception among older adults.

Indeed, the prevalence of neck pain is significant among older adults [33]. This painful context leads to a modification of the sensory proprioceptive information perceived by these individuals [34]. When pain becomes chronic (defined temporally as over 3 months), this persistent context of painful information results in reorganization of the somatosensory cortex at the cortical level. A challenge in interpreting cervical sensory information may subsequently lead to sensory reweighting, favoring other localizations, especially podal inputs. Nevertheless, this speculation should be verified through further research. This finding suggests that the sensory weighting process remains effective in older adults, notwithstanding the aging of their neuronal system, providing additional evidence of central nervous system plasticity in older adults, which is likely to play a foundational role in enhancing relearning efficiency in geriatric rehabilitation programs [35].

### Limitations

The results of this study must be interpreted in the light of our study limitations. Firstly, the sample size may be perceived as relatively small, which could limit the generalizability of our findings. Secondly, the use of a non-instrumental muscle testing scale to assess ankle strength as an inclusion criterion has been questioned. While this is a common clinical practice, the use of a dynamometer would have provided a more objective measurement. However, it is worth noting that manual muscle testing remains a widely used clinical tool for simple and inexpensive strength assessment [36].

Thirdly, the creation of a composite score to classify our elderly population lacks scientific validity. A more rigorous approach would have been to classify participants based on a validated functional test, such as the Short Physical Performance Battery. Nevertheless, the composite score is of interest as it incorporates three clinical variables commonly observed in geriatrics, including fear of falling, which is recognized as a factor impacting functional performance in older adults.

Thus, our study emphasize the importance of podal afferences for standing balance in older adults with orthopedic conditions, and suggest that optimizing cervical proprioception could be a key objective in geriatric rehabilitation to counteract the compensation by podal inputs. Further studies are necessary to verify this hypothesis of sensory reweighting and explore the potential of reducing podal dependence in older adults through interventions targeting cervical proprioceptive abilities.

## Data Availability

No datasets were generated or analysed during the current study.

## References

[1] Assländer L, Peterka RJ. Sensory reweighting dynamics in human postural control. J Neurophysiol Mai. 2014;111(9):1852–64. 10.1152/jn.00669.2013 PMC4044370 24501263

[2] Peterka RJ. Sensory integration for human balance control. In: Handbook of Clinical Neurology [Internet]. Elsevier; 2018 [cité 5 sept 2022]. pp. 27–42. Disponible sur: https://linkinghub.elsevier.com/retrieve/pii/B9780444639165000021 10.1016/B978-0-444-63916-5.00002-1 30482320

[3] Pasma JH, Engelhart D, Maier AB, Schouten AC, van der Kooij H, Meskers CGM. Changes in sensory reweighting of proprioceptive information during standing balance with age and disease. J Neurophysiol. sept 2015;30(6):3220–33. 10.1152/jn.00414.2015 PMC4686291 26424578

[4] Alberts BBGT, Selen LPJ, Medendorp WP. Age-related reweighting of visual and vestibular cues for vertical perception. J Neurophysiol 30 janv. 2019;121(4):1279–88. 10.1152/jn.00481.2018 PMC6485738 30699005

[5] Henry M, Baudry S. Age-related changes in leg proprioception: implications for postural control. J Neurophysiol août. 2019;122(2):525–38. 10.1152/jn.00067.2019 PMC6734411 31166819

[6] Sepulveda JA, Anderson AJ, Wood JM, McKendrick AM. Differential aging effects in motion perception tasks for central and peripheral vision. J Vis 20 mai. 2020;20(5):8. 10.1167/jov.20.5.8 PMC7409591 32433734

[7] Gabriel GA, Harris LR, Gnanasegaram JJ, Cushing SL, Gordon KA, Haycock BC, et al. Age-related changes to vestibular heave and pitch perception and associations with postural control. Sci Rep 19 avr. 2022;12(1):6426. 10.1038/s41598-022-09807-4 PMC9018785 35440744

[8] Poirier G, Ohayon A, Juranville A, Mourey F, Gaveau J. Deterioration, compensation and motor control processes in healthy aging, mild cognitive impairment and Alzheimer’s Disease. Geriatr 23 mars. 2021;6(1):33. 10.3390/geriatrics6010033 PMC8006018 33807008

[9] Bugnariu N, Fung J. Aging and selective sensorimotor strategies in the regulation of upright balance. J Neuroeng Rehabil 20 juin. 2007;4:19. 10.1186/1743-0003-4-19 PMC1910603 17584501

[10] Totilienė M, Uloza V, Lesauskaitė V, Damulevičienė G, Kregždytė R, Kaski D et al. Impaired Subjective Visual Vertical and Increased Visual Dependence in Older Adults With Falls. Front Aging Neurosci. 11 juin. 2021;13:667608. 10.3389/fnagi.2021.667608 PMC8232053 34177553

[11] Viseux FJF. The sensory role of the sole of the foot: review and update on clinical perspectives. Neurophysiol Clin Clin Neurophysiol févr. 2020;50(1):55–68. 10.1016/j.neucli.2019.12.003 32007381

[12] Brika M, Mourey F, Kubicki A. Sensory reweighting in frail aged adults: Are the balance deficiencies mainly compensated by visual or podal dependences? Neurosci Lett. 16 mars. 2021;747:135670. 10.1016/j.neulet.2021.135670 33516799

[13] Belloni G, Cesari M. Frailty and intrinsic capacity: two distinct but related constructs. Front Med. 2019;6:133. 10.3389/fmed.2019.00133 PMC6591451 31275941

[14] İlhan B, Bahat G, Erdoğan T, Kılıç C, Karan MA. Chronic pain: prevalent and independently associated with frailty and female gender in geriatric outpatients. Eur Geriatr Med déc. 2019;10(6):931–7. 10.1007/s41999-019-00235-8 34652781

[15] Roman de Mettelinge T, Desimpelaere P, Cambier D. Cervical mobility and cervical proprioception in relation to fall risk among older adults: a prospective cohort study. Eur Geriatr Med juin. 2023;14(3):447–53. 10.1007/s41999-023-00785-y PMC10260694 37119446

[16] Bunzeck N, Steiger TK, Krämer UM, Luedtke K, Marshall L, Obleser J, et al. Trajectories and contributing factors of neural compensation in healthy and pathological aging. Neurosci Biobehav Rev janv. 2024;156:105489. 10.1016/j.neubiorev.2023.105489 38040075

[17] Kempen GIJM, Yardley L, van Haastregt JCM, Zijlstra GAR, Beyer N, Hauer K, et al. The short FES-I: a shortened version of the falls efficacy scale-international to assess fear of falling. Age Ageing janv. 2008;37(1):45–50. 10.1093/ageing/afm157 18032400

[18] Konor MM, Morton S, Eckerson JM, Grindstaff TL. Reliability of three measures of ankle dorsiflexion range of motion. Int J Sports Phys Ther juin. 2012;7(3):279–87. PMC3362988 22666642

[19] Mehmet H, Yang AWH, Robinson SR. Measurement of hand grip strength in the elderly: a scoping review with recommendations. J Bodyw Mov Ther janv. 2020;24(1):235–43. 10.1016/j.jbmt.2019.05.029 31987550

[20] Mehmet H, Robinson SR, Yang AWH. Assessment of Gait Speed in Older Adults. J Geriatr Phys Ther. 2001. 2020;43(1):42–52. 10.1519/JPT.0000000000000224 30720555

[21] Michiels S, De Hertogh W, Truijen S, November D, Wuyts F, Van De Heyning P. The assessment of cervical sensory motor control: a systematic review focusing on measuring methods and their clinimetric characteristics. Gait Posture mai. 2013;38(1):1–7. 10.1016/j.gaitpost.2012.10.007 23153836

[22] Alahmari K, Reddy RS, Silvian P, Ahmad I, Nagaraj V, Mahtab M. Intra- and inter-rater reliability of neutral head position and target head position tests in patients with and without neck pain. Braz J Phys Ther. 2017;21(4):259–67. 28558952 10.1016/j.bjpt.2017.05.003 PMC5537472

[23] Roren A, Mayoux-Benhamou MA, Fayad F, Poiraudeau S, Lantz D, Revel M. Comparison of visual and ultrasound based techniques to measure head repositioning in healthy and neck-pain subjects. Man Ther juin. 2009;14(3):270–7. 10.1016/j.math.2008.03.002 18514016

[24] Hill R, Jensen P, Baardsen T, Kulvik K, Jull G, Treleaven J. Head repositioning accuracy to neutral: a comparative study of error calculation. Man Ther févr. 2009;14(1):110–4. 10.1016/j.math.2008.02.008 18502679

[25] Anson E, Bigelow RT, Studenski S, Deshpande N, Agrawal Y. Failure on the foam eyes closed test of standing Balance Associated with reduced semicircular canal function in healthy older adults. Ear Hear. 2019;40(2):340–4. 29894381 10.1097/AUD.0000000000000619 PMC6289873

[26] Hupfeld KE, McGregor HR, Hass CJ, Pasternak O, Seidler RD. Sensory system-specific associations between brain structure and balance. Neurobiol Aging Nov. 2022;119:102–16. 10.1016/j.neurobiolaging.2022.07.013 PMC9728121 36030560

[27] Vuillerme N, Pinsault N, Bouvier B. Cervical joint position sense is impaired in older adults. Aging Clin Exp Res août. 2008;20(4):355–8. 10.1007/BF03324868 18852550

[28] Alahmari KA, Reddy RS, Silvian PS, Ahmad I, Kakaraparthi VN, Alam MM. Association of age on cervical joint position error. J Adv Res Mai. 2017;8(3):201–7. 10.1016/j.jare.2017.01.001 PMC5292654 28203459

[29] Qu N, Tian H, De Martino E, Zhang B. Neck Pain: do we know enough about the Sensorimotor Control System? Front Comput Neurosci. 2022;16:946514. 35910451 10.3389/fncom.2022.946514 PMC9337601

[30] Reddy RS, Alkhamis BA, Kirmani JA, Uddin S, Ahamed WM, Ahmad F et al. Age-Related Decline in Cervical Proprioception and Its Correlation with Functional Mobility and Limits of Stability Assessed Using Computerized Posturography: A Cross-Sectional Study Comparing Older (65 + Years) and Younger Adults. Healthc Basel Switz. 3 juill. 2023;11(13):1924. 10.3390/healthcare11131924 PMC10340456 37444758

[31] Raizah A, Reddy RS, Alshahrani MS, Gautam AP, Alkhamis BA, Kakaraparthi VN et al. A Cross-Sectional Study on Mediating Effect of Chronic Pain on the Relationship between Cervical Proprioception and Functional Balance in Elderly Individuals with Chronic Neck Pain: Mediation Analysis Study. J Clin Med. 26 avr. 2023;12(9):3140. 10.3390/jcm12093140 PMC10179428 37176581

[32] Quek J, Treleaven J, Clark RA, Brauer SG. An exploratory study examining factors underpinning postural instability in older adults with idiopathic neck pain. Gait Posture févr. 2018;60:93–8. 10.1016/j.gaitpost.2017.11.016 29175640

[33] Kazeminasab S, Nejadghaderi SA, Amiri P, Pourfathi H, Araj-Khodaei M, Sullman MJM, et al. Neck pain: global epidemiology, trends and risk factors. BMC Musculoskelet Disord 3 janv. 2022;23(1):26. 10.1186/s12891-021-04957-4 PMC8725362 34980079

[34] Stanton TR, Leake HB, Chalmers KJ, Moseley GL. Evidence of impaired proprioception in Chronic, Idiopathic Neck Pain: systematic review and Meta-analysis. Phys Ther juin. 2016;96(6):876–87. 10.2522/ptj.20150241 PMC4897597 26472296

[35] Cristini J, Parwanta Z, De Las Heras B, Medina-Rincon A, Paquette C, Doyon J, et al. Motor memory consolidation deficits in Parkinson’s Disease: a systematic review with Meta-analysis. J Park Dis. 2023;13(6):865–92. 10.3233/JPD-230038 PMC10578244 37458048

[36] Bohannon RW. Manual muscle testing: does it meet the standards of an adequate screening test? Clin Rehabil Sept. 2005;19(6):662–7. 10.1191/0269215505cr873oa 16180603

